# LAG3 as a Tumor Suppressor and Immune Regulator in Cervical Cancer: From Functional Validation to Therapeutic Strategy

**DOI:** 10.1002/cam4.71278

**Published:** 2025-09-30

**Authors:** Shijie Yao, Siming Chen, Shimeng Wan, Anjin Wang, Ziyan Liang, Xuelian Liu, Yang Gao, Hongbing Cai

**Affiliations:** ^1^ Department of Gynecological Oncology Zhongnan Hospital of Wuhan University Wuhan China; ^2^ Department of Urology Zhongnan Hospital of Wuhan University Wuhan China

**Keywords:** chemotherapy response, GEO, pyroptosis, TCGA, tumor immune microenvironment

## Abstract

**Background:**

Cervical cancer remains a significant health burden for women worldwide, with persistent high‐risk HPV infection being a major etiological factor. Despite treatment advances, prognosis for recurrent or metastatic disease remains poor. Pyroptosis, a form of programmed cell death, plays a dual role in tumor immunity, but its implications in cervical cancer are not fully elucidated. This study aims to systematically characterize pyroptosis‐related genes (PRGs) in cervical cancer and explore their prognostic and therapeutic relevance.

**Methods:**

Multi‐omics data from TCGA and GEO databases were integrated to analyze genetic variations, expression patterns, and prognostic significance of 52 PRGs in cervical cancer. Consensus clustering was used to identify PRG subtypes. A prognostic risk score model was constructed using LASSO‐Cox regression based on differentially expressed genes (DEGs). Functional validation was performed via in vitro and in vivo experiments, including Western blot, CCK‐8, colony formation, transwell assays, and a subcutaneous tumor model. Single‐cell RNA sequencing data (GSE171894, GSE168652) were analyzed to explore LAG3 expression in the tumor immune microenvironment.

**Results:**

Two distinct PRG subtypes were identified, with subtype A showing immune activation features. A five‐gene prognostic signature (GNAZ, LAG3, IL‐1β, CA2, SPRR3) effectively stratified patients into high‐ and low‐risk groups. Low LAG3 expression was associated with poor prognosis. Functional experiments demonstrated that LAG3 overexpression suppressed cervical cancer cell proliferation, migration, and tumor growth, while its knockdown promoted malignant phenotypes. Single‐cell analysis revealed high LAG3 expression in Treg and CD8⁺ T cells, suggesting its role in immune regulation.

**Conclusion:**

This study establishes a novel PRG‐based prognostic model and highlights LAG3 as a key tumor suppressor and immune regulator in cervical cancer. These findings provide insights into the interplay between pyroptosis and antitumor immunity, supporting LAG3 as a potential therapeutic target for cervical cancer immunotherapy.

## Graphical abstract

Studies have found that the molecular typing of pyroptostic‐related genes (PRGs) in cervical cancer can predict prognosis, and the GNAZ/LAG3/IL1B/CA2/SPRR3 risk scoring model has been constructed. Experiments have confirmed that LAG3 inhibits tumor progression by regulating Treg and CD8^+^ T cells, providing a new target for immunotherapy.

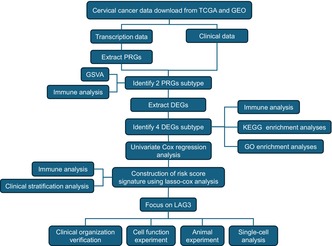

## 1 Introduction

Cervical cancer is one of the most common malignant tumors in the world [1, 2]. It is one of the leading causes of cancer‐related deaths in women, with about 311,000 women dying of cervical cancer in 2018 [3]. The disease poses a serious threat to women's physical health and profoundly impacts their emotional and psychological well‐being [4, 5, 6]. The treatment plan for cervical cancer is mainly based on the clinical stage of the disease. In the early stage, the treatment usually includes surgery combined with chemotherapy and radiotherapy [7]. While in the advanced stage or when metastasis occurs, the treatment is mainly based on chemotherapy and radiotherapy, and palliative care can be considered to relieve symptoms when necessary [8]. In addition, immunotherapy has brought new therapeutic hope to patients with cervical cancer [9, 10]. However, due to the heterogeneity among patients, a portion of cervical cancer patients shows resistance to immunotherapy [11, 12]. Therefore, it is important to explore new therapeutic targets to improve the prognosis of cervical cancer patients.

Pyroptosis is a type of programmed cell death (PCD) activated by inflammatory vesicles [13] and mediated primarily by cysteine aspartate proteases [14]. This process was first identified in 1992 in association with phagocytosis by macrophages [15] and plays a key role in the clearance of bacterial and viral infections [16]. Gasdermin D (GSDMD) and Gasdermin E (GSDME) are GSDMs that play an important role in pyroptosis [17, 18]. GSDMD is cleaved by Caspase‐1, which is activated to expose its N‐terminal structural domain (GSDMD‐N), thereby initiating pyroptosis [19, 20]. In addition, Caspase‐3 specifically cleaves GSDME, generating its N‐terminal structural domain (GSDME‐N), a process that increases the permeability of the mitochondrial membrane, prompting the release of Cyt c, which further activates cellular focal death [21, 22]. Tumorigenesis and progression have been suggested to be closely linked to the regulation of Gasdermin proteins, which act by inducing pyroptosis [18, 23, 24, 25]. In addition, pyroptosis acts as a natural immune defense mechanism involved in regulating the tumor immune microenvironment (TIME) [26]. However, the specific role of cellular pyroptosis in the TIME of cervical cancer is not fully understood, and in‐depth systematic studies are lacking.

This study comprehensively analyzed mutation profiles, copy number variations (CNVs), and expression patterns of 52 pyroptosis‐related genes (PRGs) using multi‐center datasets (TCGA and GEO). Molecular subtyping via consensus clustering revealed distinct immune infiltration characteristics. A prognostic prediction model was constructed by combining key genes (GNAZ, LAG3, IL‐1β, CA2, and SPRR3) and clinicopathologic parameters. Functional experiments demonstrated that LAG3 inhibits tumor proliferation and migration, while single‐cell sequencing identified its predominant expression in Treg and CD8^+^ T cells, implicating its role in immune regulation. These findings enhance our understanding of PRGs in cervical cancer and offer new directions for prognostic evaluation and immunotherapy.

## 2 Result

### 2.1 Investigation of the Expression and Genetic Variability of Pyroptosis‐Related Genes in Cervical Cancer

**FIGURE 1:**
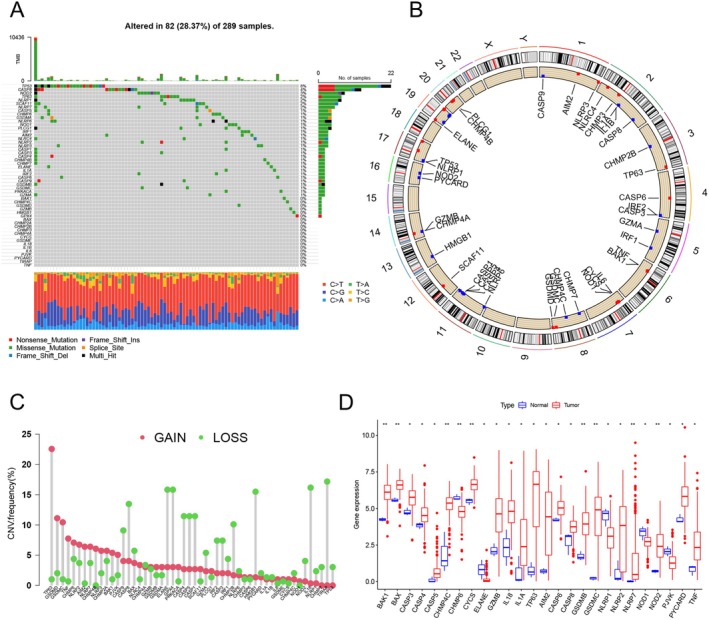
Investigation of the expression and genetic variability of pyroptosis‐related genes in cervical cancer. (A) Mutation frequencies of 52 PRGs in the TCGA cervical cancer dataset. Each column represents an individual patient, while each row corresponds to a specific PRG. (B) The chromosomal localization of PRGs is shown, with red dots indicating amplifications and blue dots representing deletions. (C) The frequencies of CNVs for various PRGs in the TCGA cohort are represented by the height of the columns, reflecting the percentage of variation observed. (D) The expression levels of PRGs differ between normal tissues and cervical cancer tissues. Blue boxes represent normal cervical samples, while red boxes represent cervical cancer samples. Asterisks indicate statistical significance (*p < 0.05; **p < 0.01).

Using the TCGA dataset, we analyzed 52 PRGs to assess the occurrence of CNVs and somatic mutations in cervical cancer (Table S1). Our findings revealed that mutations were present in 82 out of 289 samples (28.37%) (Figure 1A). Among these mutations, missense mutations were the most common type, with C > T mutations being the predominant form of single‐nucleotide polymorphisms (SNPs). At the genetic level, a total of 38 PRGs were identified as having mutations. TP53 had the highest mutation frequency at 8%, followed by CASP8, which had a mutation frequency of 4%. We also observed changes in the CNV‐related genes across chromosomes (Figure 1B). The frequency of CNV alterations in the 52 PRGs indicated a high prevalence of CNV modifications. TP63 exhibited the highest frequency of CNV amplification, while TIRAP had the most frequent CNV deletion (Figure 1C). Next, we compared the expression levels of 52 PRGs in 3 normal tissues and 306 tumor tissues from TCGA, and the analysis revealed a total of 26 differentially expressed PRGs. Among them, 21 PRGs were expressed at higher levels in tumor tissues compared to normal tissues, while the remaining 5 PRGs showed lower expression in tumor tissues than in normal tissues (Figure 1D). To further investigate the impact of PRGs on cervical cancer, we integrated the TCGA and GSE30759 datasets to obtain PRG expression levels, resulting in a set of 34 PRGs that had a significant impact on the prognosis of cervical cancer, and we constructed a PRG correlation network to examine the interactions among these PRGs (Figure S1A). We explored the potential impact of these 34 PRGs on cervical cancer prognosis using Kaplan–Meier analysis and found that 22 PRGs showed significant variations in overall survival (OS). Of these, 11 PRGs were positively associated with prognosis (Figure S1B–L), while the remaining 11 PRGs were negatively correlated with prognosis (Figure S2A–K).

### 2.2 Molecular Patterning and Genotyping Identification of Pyroptosis‐Related Genes

**FIGURE 2:**
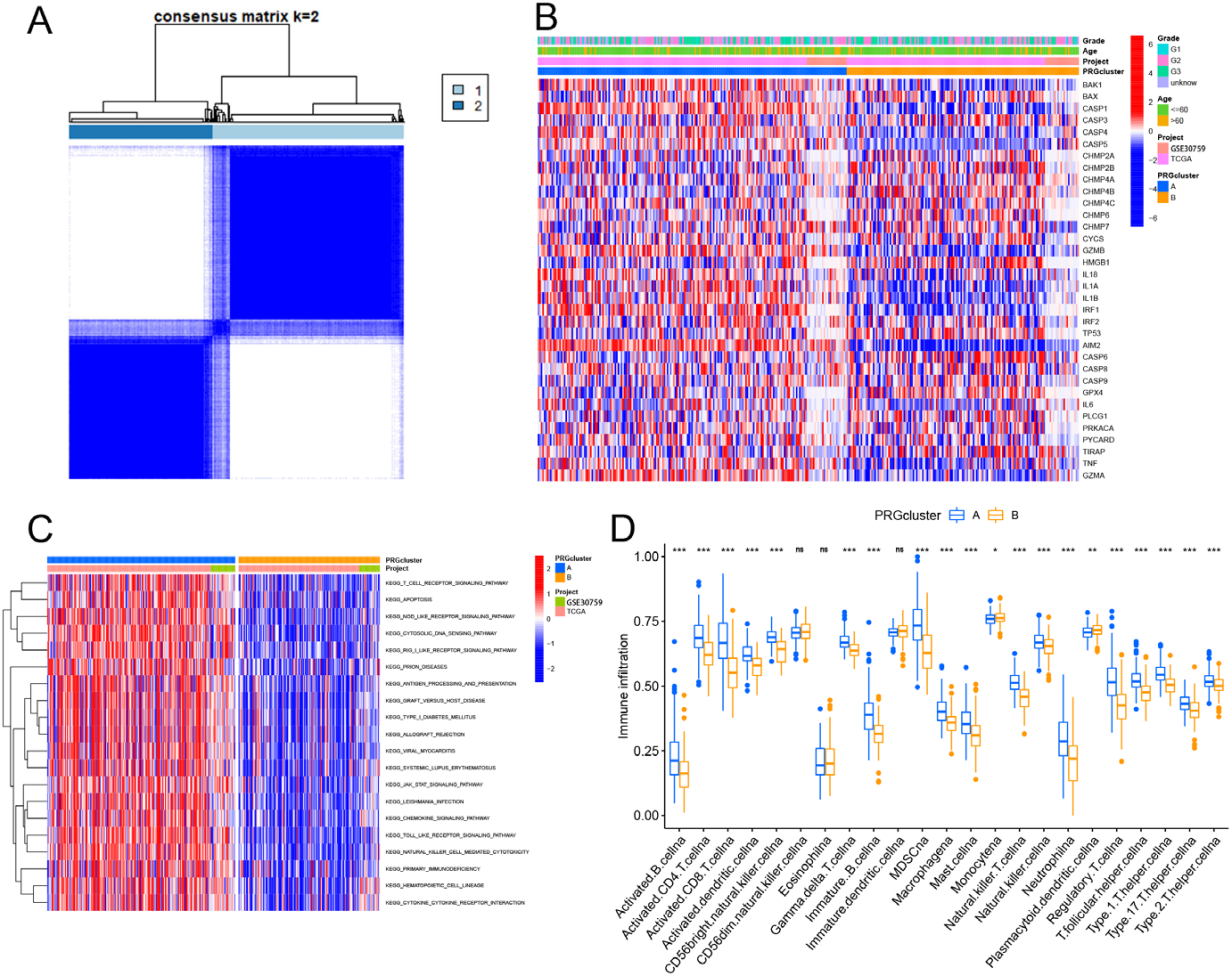
Molecular patterning and genotyping identification of pyroptosis‐related genes. (A) The TCGA and GEO datasets were used to create a consensus clustering matrix for cervical cancer patients (*K* = 2). (B) A heatmap illustrating the distribution of gene expression and clinical traits across the two PRG clusters. Clinical traits included: Patient age and histologic grading. (C) A heatmap visualizing the GSVA‐analyzed biological processes, highlighting bioactive pathways associated with different PRG clusters. Red represented activated pathways and blue represented inhibited pathways. (D) Significant differences were observed in the expression of immune cells between the two PRG clusters. (ns, not statistically significant; *p < 0.05; **p < 0.01; ***p < 0.001).

To better understand the association between PRG expression and cervical cancer, we used the “ConsensusClusterPlus” program to categorize the TCGA cervical cancer cohort based on 34 PRG expression levels that significantly affected prognosis. When *K* = 2, the intra‐group correlation was highest, while the inter‐group correlation was lowest (Figure 2A; Figure S3A–I). The PRG clusters of the two groups were distinguishable, as shown by the PCA results (Figure S3K). A heatmap displayed the distribution of subtypes within the two PRG clusters (Figure 2B). We compared the OS of the two PRG clusters and found no significant differences between subgroups. However, the median survival time of PRG cluster B was slightly better than that of cluster A (Figure S3J). To explore the biological differences between the two PRG clusters, we conducted a GSVA enrichment analysis. The results indicated that PRG cluster A exhibited an immune activation state, as indicated by the enrichment of immune‐related pathways, including KEGG_T_CELL_RECEPTOR_SIGNALING_PATHWAY, KEGG_ANTIGEN_PROCESSING_AND_PRESENTATION, and KEGG_ALLOGRAFT_REJECTION. In addition, several signaling‐related pathways, such as KEGG_CYTOSOLIC_DNA_SENSING_PATHWAY and KEGG_JAK_STAT_SIGNALING_PATHWAY, were found to be active. In contrast, immune‐related pathways were suppressed in PRG cluster B (Figure 2C). We also examined the distribution of 23 immune cell types in the PRG clusters to analyze their immunological characteristics. PRG cluster A showed a higher infiltration of immune cells compared to PRG cluster B (Figure 2D).

### 2.3 Prognostic Landscape of Pyroptosis‐Related Genes Based on DEGs

**FIGURE 3:**
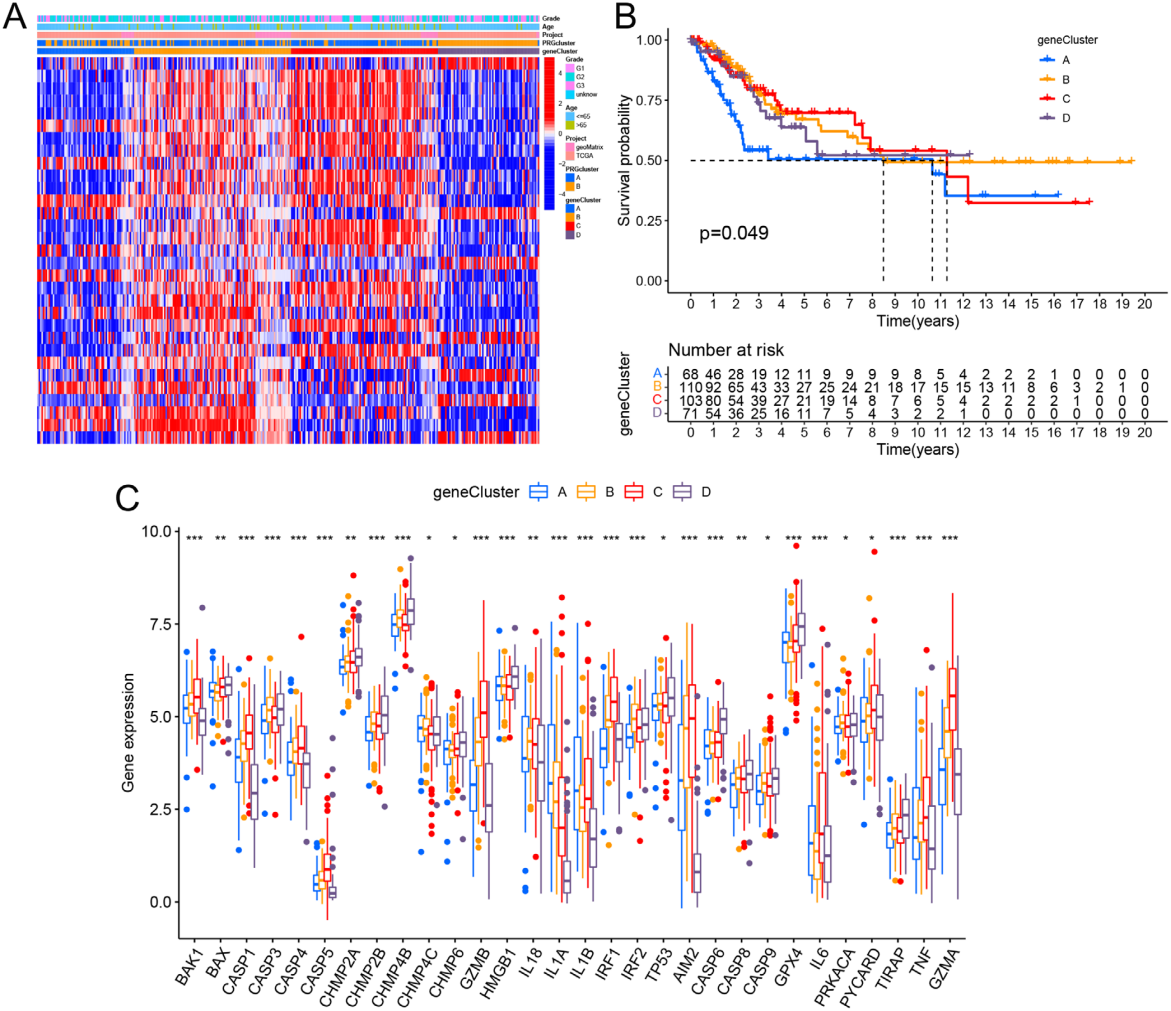
Prognostic landscape of pyroptosis‐related genes based on DEGs. (A) A heatmap showing gene expression and the distribution of clinical traits across the four DEG clusters. Clinical traits included: Patient age and histologic grading. (B) Kaplan–Meier survival analysis for OS among the four DEG clusters (*p* = 0.049) (Cluster A: Blue; B: Orange; C: Red; D: Purple). (C) Variation in the expression levels of 30 PRGs across the four DEG clusters (Cluster A: Blue; B: Orange; C: Red; D: Purple) (*p < 0.05; **p < 0.01; ***p < 0.001).

To explore the gene cluster differences between the two PRG clusters, we extracted 324 differentially expressed genes (DEGs) from the two PRG clusters (Table S2). The functional status of these 324 DEGs was assessed through KEGG and GO enrichment analyses. The biological processes (BPs) were primarily associated with epidermis development and the regulation of peptidase activity. The cellular components (CCs) showed a high abundance of these genes on the external side of the plasma membrane, the cell–cell junction, and the apical part of the cell. In terms of molecular functions (MFs), the target genes influenced receptor ligand activity, peptidase regulator activity, and cytokine activity (Figure S4A). According to the KEGG analysis, these genes were linked to cytokine‐cytokine receptor interaction, Staphylococcus aureus infection, and cell adhesion molecules (Figure S4B). To further investigate the correlation between the 324 DEGs and cervical cancer prognosis, we performed univariate Cox regression analysis, which identified 31 DEGs associated with cervical cancer prognosis (Table S3). Using these 31 DEGs for unsupervised clustering of all samples, we found that *K* = 4 provided the greatest intra‐group correlation while maintaining a low inter‐group correlation (Figure S5A–J). Principal component analysis (PCA) revealed a high degree of independence between the different subgroups (Figure S5K). A heatmap showed the different gene expression patterns across the DEG clusters (Figure 3A). Survival analysis based on the clustering of the four DEG clusters showed that DEG cluster A exhibited the worst OS, while DEG cluster C exhibited the best OS (*p* = 0.049) (Figure 3B). We also examined the differential expression of 52 PRGs across the four DEG clusters; the result revealed that 30 PRGs exhibited significant expression variations across these DEG clusters (Figure 3C).

### 2.4 Construction and Validation of Prognostic Model

**FIGURE 4:**
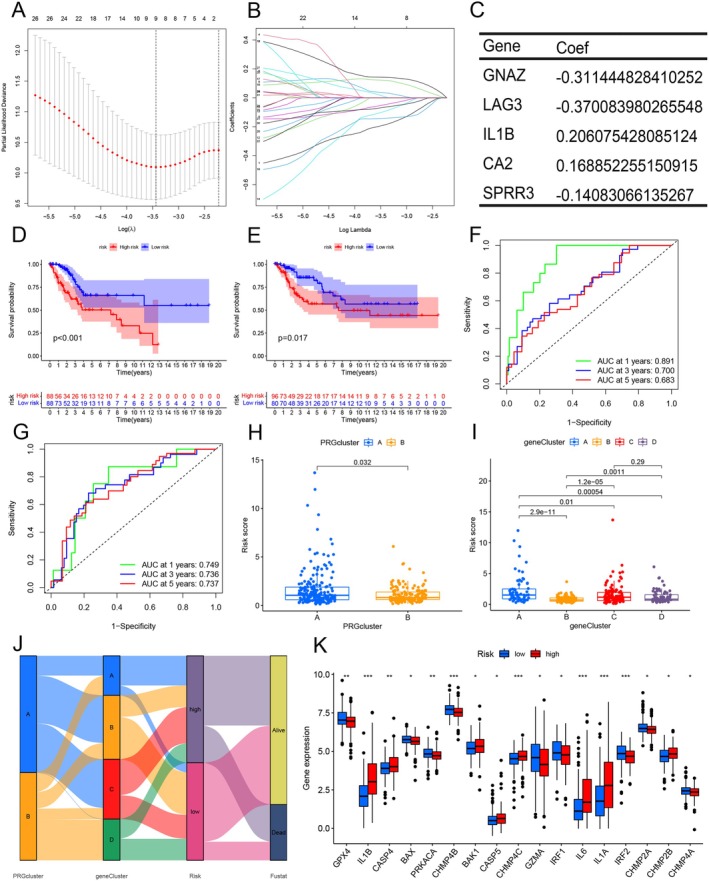
Construction and validation of prognostic model. (A) LASSO regression was performed on a set of 5 DEGs associated with OS. (B) Cross‐validation was used to adjust parameter selection in LASSO regression. (C) 5 key DEGs with corresponding parameters involved in prognostic model construction. (D) In the training cohort, comparing the survival rates of high‐risk and low‐risk groups using a Kaplan–Meier survival analysis. (E) Performing a Kaplan–Meier survival analysis to compare the survival rates of the high‐risk and low‐risk groups in the testing cohort. (F) The ROC curves of the prediction models in the training cohort. (G) The ROC curve of the prediction model in the testing cohort. (H) Differential expression of risk scores between 2 subgroups based on consensus clustering of PRGs. (I) Differential expression of risk scores between 4 subgroups based on DEG consensus cluster typing. (J) Sankey diagram showing the distribution of PRGs clusters, DEG clusters, risk scores, and survival status (fustat). (K) There was a disparity in the PRGs between the high‐risk score groups and the low‐risk score groups (*p < 0.05; **p < 0.01; ***p < 0.001).

We randomly divided the cervical cancer dataset into training and testing groups using Stratified Random Sampling (SRS) and applied a LASSO‐Cox regression model with a minimum λ (Figure 4A,B). Five key genes were selected from the 31 DEGs to construct a risk score model, incorporating clinical variables. The resulting model is as follows: Risk Score = −0.3114 × GNAZ—0.3701 × LAG3–0.2061 × IL‐1β + 0.1689 × CA2–0.1408 × SPRR3 (Figure 4C). Scatter plots and heat maps showed that patients in the high‐risk group had more deaths (Figure S6A–I). Kaplan–Meier curves consistently demonstrated that patients in the high‐risk group had worse OS (Figure 4D,E; Figure S6J). ROC curve analysis showed that the predictive model was effective in forecasting patient prognosis, with AUC values of 0.891, 0.700, and 0.683 for 1‐year, 3‐year, and 5‐year survival, respectively, in the training group (Figure 4F). Similar results were observed in the overall sample group and the testing group (Figure 4G; Figure S6K). These findings suggest that the risk score may serve as a reliable independent predictor. To assess the impact of the risk score on cervical cancer, we examined its distribution across different consensus clustering samples; the results showed that PRG cluster A had a higher risk score (Figure 4H). Apart from the risk scores between clusters C and D in the DEG cluster, significant variations in risk scores were observed across the other groups (Figure 4I). The Sankey diagram visually displayed the distribution of PRG clustering and DEG clustering with risk score and survival status, validating the biological consistency and prognostic relevance of the multilevel hierarchical models (Figure 4J). We also analyzed the differences in the expression of PRGs across risk scores. The results revealed significant differences in the expression of 17 PRGs between the high‐ and low‐risk groups. Nine PRGs were more highly expressed in the low‐risk group (GPX4, BAX, PRKACA, CHMP4B, GZMA, IRF1, IRF2, CHMP2A, CHMP4A), while the expression of the remaining 8 PRGs was higher in the high‐risk group (IL‐1β, CASP4, BAK1, CASP5, CHMP4C, IL6, IL1A, CHMP2B) (Figure 4K). We integrated clinicopathological data and developed a nomogram using grade, age, and risk score as key factors (Figure S7A). A numerical value was assigned to each clinical feature, and the prediction score was computed by aggregating the scores of different attributes to estimate the patient's prognosis. The calibration curve demonstrated a high degree of concordance between the nomogram's predictions and actual OS outcomes (Figure S7B).

### 2.5 Risk Score and Immune Microenvironment Assessment and Immunotherapy Prediction

**FIGURE 5:**
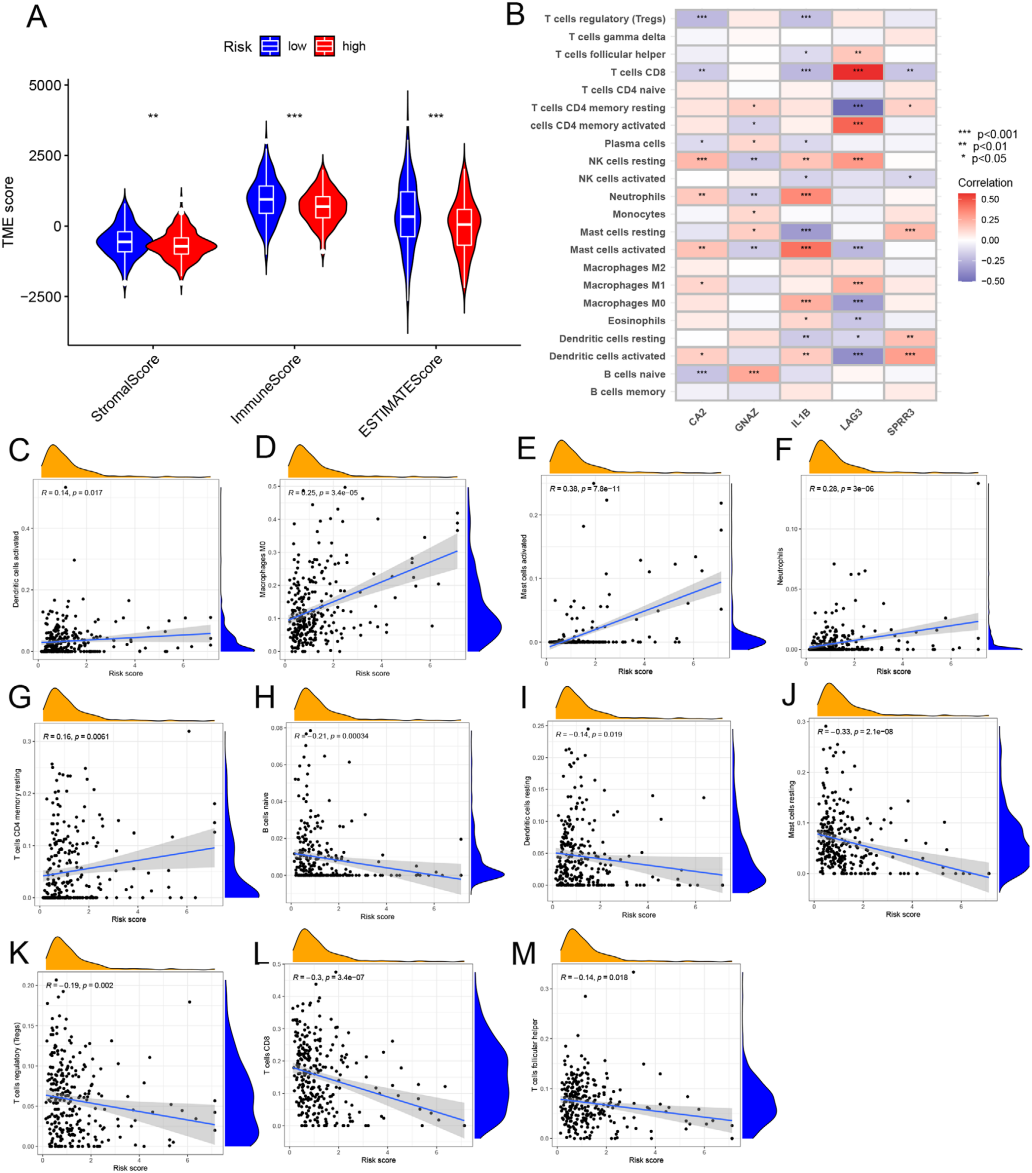
Risk score and immune microenvironment assessment and immunotherapy prediction. (A) TME scores vary between high‐risk and low‐risk groups, including the stromal, immune, and estimate scores. (B) Heatmap showing the correlation between the expression of 5 key DEGs involved in risk score construction and immune cell infiltration. (C–M) Correlation between risk score and immune cell infiltration (*p < 0.05; **p < 0.01; ***p < 0.001).

Studies have shown a correlation between pyroptosis and immune cell infiltration [27]. In this study, we assessed the total number of immune cells in each sample and evaluated the state of the tumor immune microenvironment. We also explored the relationship between key DEGs and immune cells to understand the role of these DEGs in constructing prognostic models within the TIME. The results indicated that stromal scores, immune scores, and estimate scores differed across the different risk score subgroups (Figure 5A). In the heatmap, the relationships between the expression of 5 key DEGs and the 22 types of immune cell infiltration were illustrated. CD8^+^ T cells and LAG3 showed a positive correlation, while naive B cells and CA2 had a negative association (Figure 5B). We calculated the differences in immune cell infiltration levels across risk score subgroups. 11 immune cell types exhibited significant correlations with risk scores, with activated dendritic cells, M0 macrophages, activated mast cells, neutrophils, and resting memory CD4^+^ T cells being positively correlated with risk scores (Figure 5C–G). In contrast, naive B cells, resting dendritic cells, resting mast cells, regulatory T cells (Tregs), CD8^+^ T cells, and follicular helper T cells showed a negative correlation with risk scores (Figure 5H–M). To evaluate the sensitivity to chemotherapeutic agents in different risk score subgroups, we calculated the IC50 values for various drugs. The IC50 values for 39 immunotherapy drugs showed a significant correlation with risk score, suggesting that the risk score could be a valuable predictor for immunotherapy response (Figure S8).

### 2.6 Identification of Key Genes and Determination of Gene Function

**FIGURE 6:**
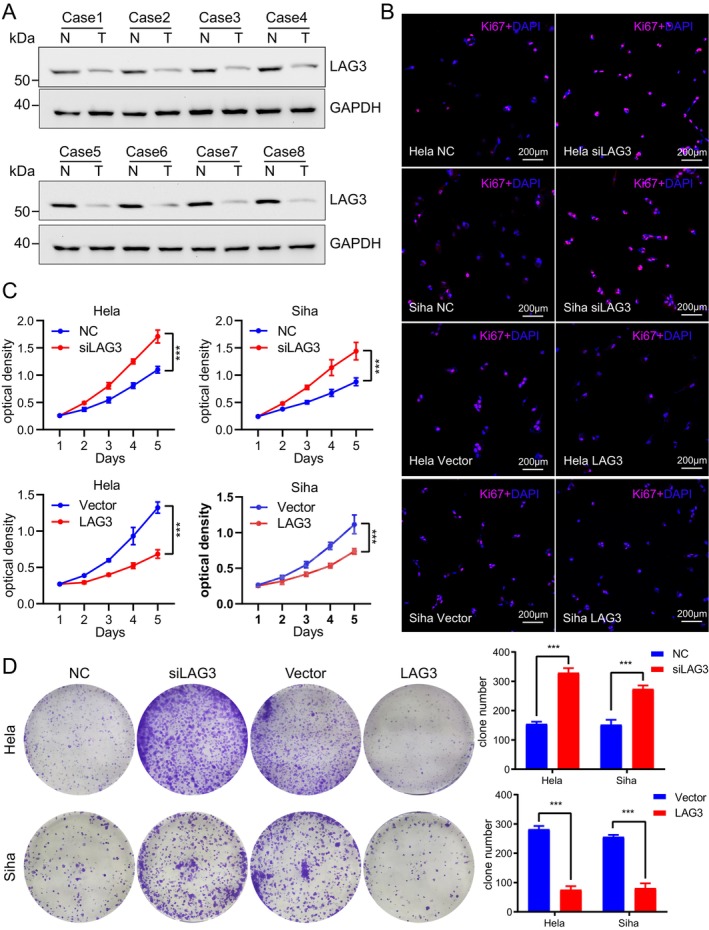
LAG3 affects the proliferative capacity of cervical cancer cells. (A) The protein level of LAG3 was confirmed in 8 cervical cancer tissues and corresponding paraneoplastic tissues using Western blot analysis, N stands for cervical tissue, T stands for cervical cancer tissue. (B) Immunofluorescence was used to assess the proliferative ability of Siha cells and Hela cells after LAG3 knockdown and overexpression. (C) The proliferative ability of Siha cells and Hela cells were evaluated by CCK8 assay after LAG3 knockdown or overexpression. Data are presented as mean values ± SD, with *n* = 6 technical independent experiments. (D) Colony formation assays of Siha cells and Hela cells proliferation capacity after LAG3 knockdown or overexpression (left panel), followed by statistical analysis (right panel). Data are presented as mean values ± SD, with *n* = 3 biological independent experiments. Statistical significance was determined by two‐tailed unpaired Student's *t*‐test (C, D). (**p < 0.01; ***p < 0.001).

To assess the significance of five key genes in cervical cancer, we used the UALCAN website to analyze data on their association with OS. The results revealed that low expression of LAG3 was linked to a poor prognosis in cervical cancer (*p* = 0.0027), while high expression of IL‐1β was also associated with poor prognosis (*p* = 0.0014). However, the expression of CA2, GNAZ, and SPRR3 did not appear to affect cervical cancer prognosis (Figure S9A–E). Previous studies have suggested that altering IL‐1β expression may influence the proliferation and migration of cervical cancer cells [28]. As a result, we decided to focus on LAG3 for further investigation. Using western blotting, we confirmed that LAG3 expression was reduced in cervical cancer tissues (Figure 6A; Table S4).

**FIGURE 7:**
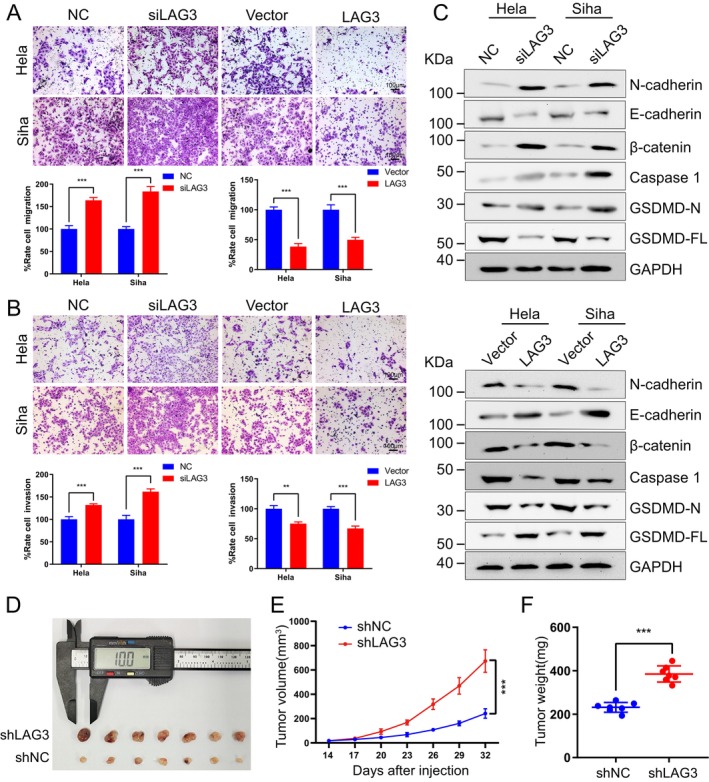
LAG3 modulates migration, invasion, and tumor growth in cervical cancer. (A) Transwell assay were used to assess the migratory ability of Siha cells and Hela cells after LAG3 knockdown or overexpression (upper panel), followed by statistical analysis (lower panel). Data are presented as mean values ± SD, with *n* = 3 biological independent experiments. (B) Transwells assay were used to evaluate the invasive capacity of Siha cells and Hela cells after LAG3 knockdown or overexpression (upper panel), followed by statistical analysis (lower panel). Data are presented as mean values ± SD, with *n* = 3 biological independent experiments. (C) Western blot assay was used to detect the expression levels of some proteins, including N‐cadherin, E‐cadherin, β‐catenin, Caspase 1, GSDMD‐N, and GSDMD‐FL and GAPDH, after knockdown or overexpression of LAG3 in Hela and Siha cells. (D) Anatomical diagram of subcutaneous tumor tissue in different groups. (E) Statistical plot of subcutaneous tumor growth volume between groups. Data are presented as mean values ± SD, with *n* = 7 biological independent experiments. (F) Weight of subcutaneous tumors affected by *LAG3* knockdown. Data are presented as mean values ± SD, with *n* = 7 biological independent experiments. Statistical significance was determined by two‐tailed unpaired Student's *t*‐test (A, B, E, F). (**p < 0.01; ***p < 0.001).

To investigate the effect of LAG3 on cervical cancer cell biology, we first optimized the dose–response of *siLAG3* transfection in Hela and Siha cells and verified the overexpression efficiency of the LAG3 plasmid. Based on Western Blot and RT‐qPCR results, the optimal *siLAG3* concentration (50 nM) was determined and used for subsequent cell function experiments. In addition, the LAG3 plasmid showed significant overexpression efficiency (Figure S10A,B). Immunofluorescence experiments revealed that increased LAG3 expression reduced the proliferative capacity of cervical cancer cells, while knockdown of *LAG3* enhanced their proliferative capacity (Figure 6B). Similar results were obtained using the CCK8 and colony formation assays (Figure 6C,D). We then performed transwell assays, which showed that *LAG3* knockdown increased the migratory and invasive capabilities of cervical cancer cell lines, while LAG3 overexpression reduced these capabilities (Figure 7A,B). Additionally, we measured the expression levels of N‐cadherin, E‐cadherin, β‐catenin, Caspase 1, GSDMD‐N, and GSDMD‐FL in Hela and Siha cells. When *LAG3* was knocked down, the expression of all proteins, except E‐cadherin and GSDMD‐FL, was upregulated (Figure 7C). The opposite occurred when LAG3 was overexpressed. To investigate the role of LAG3 in cervical cancer proliferation in vivo, we established a subcutaneous tumor model. The results showed that *LAG3* knockdown significantly increased the weight and volume of subcutaneous tumors compared to controls (Figure 7D–F). Taken together, these findings support the conclusion that LAG3 inhibits the proliferation of cervical cancer cells.

### 2.7 Immune Cell Potential Value of LAG3

**FIGURE 8:**
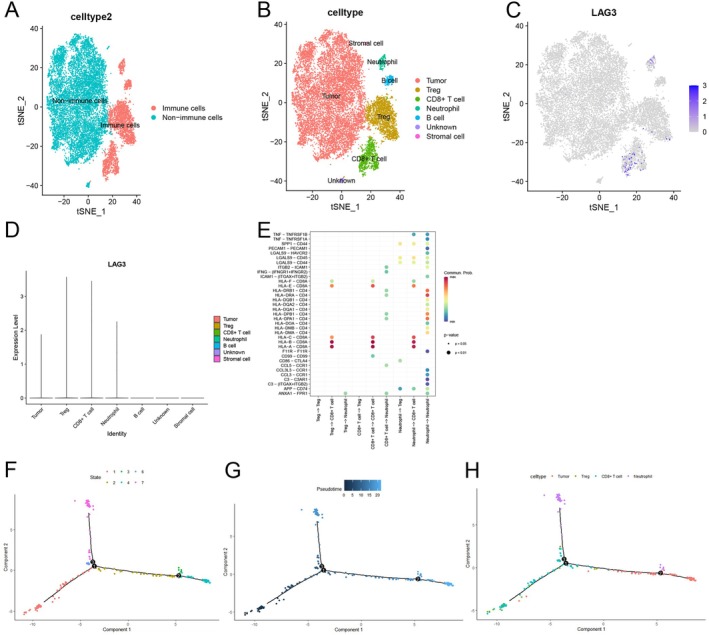
Localization, manifestation, and intercellular signaling of LAG3. (A) Overall distribution of immune and non‐immune cells. (B) Cells are categorized into seven kinds using cell‐specific marker genes, which include tumor cells, Treg cells, CD8^+^ T cells, neutrophils, B cells, stromal cells, and unknown. (C) Cellular distribution of LAG3 across several cell types. (D) Quantification of LAG3 expression in various cell types. (E) Cellular communication between LAG3‐localized cell populations. (F) The temporal differentiation pathway of immune cells, which gives rise to a total of three branches and three temporal differentiation nodes. (G) Greater color intensity corresponds to earlier differentiation time. (H) Positions of CD8^+^ T cells, Treg, Neutrophil, and tumor cells in the temporal differentiation trajectory.

To further explore the role of LAG3 in the immune microenvironment of cervical cancer, we obtained the single‐cell datasets GSE171894 and GSE168652 from the GEO database. These datasets were combined to examine the transcriptional patterns in cervical cancer. The combined dataset included one sample of normal cervical tissue and five cervical cancer tissue samples. After removing low‐quality cells, we used t‐SNE to depict the overall distribution of the cells visually. We observed noticeable heterogeneity between immune and non‐immune cells (Figure 8A). The cells were classified into seven main types: tumor, Treg, CD8^+^ T cell, neutrophil, B cell, stromal cell, and unknown. This classification was based on the analysis of differentially expressed genes and cell‐specific marker genes for each cluster (Figure 8B). In the atlas, we detected LAG3 expression in several cell groups, including Treg cells, CD8^+^ T cells, neutrophils, and tumor cells (Figure 8C). Notably, the highest levels of LAG3 expression were found in Treg cells and CD8^+^ T cells (Figure 8D). The cellular communication analysis revealed robust and concentrated intercellular signaling between the cell populations mediated by LAG3 (Figure 8E). The Monocle2 tool was used to examine the sequential arrangement of cell populations regulated by LAG3. This analysis identified three distinct cell branches and three temporal differentiation nodes (Figure 8F–H). Finally, we conducted a functional enrichment analysis, which showed that LAG3 is primarily involved in T cell activation and antigen presentation, as indicated by the GO and KEGG results (Figure S11A,B).

## 3 Discussion

### 3.1 Role of Pyroptosis and Immune Modulation in Cervical Cancer

Cervical cancer is a malignancy that specifically targets the female reproductive system. The primary cause of almost 90% of cervical malignancies is a chronic infection of the human papillomavirus (HPV) [29], HPV can establish itself in the basal layer of cervical keratinocytes, and its ability to evade the immune system is a significant factor in its persistence and integration into the host cell's genetic material. This eventually results in the progression of cervical cancer [30, 31]. The treatment of cervical cancer has made great progress, but there are still certain challenges. Individuals with recurrent and metastatic cervical cancer still have a dismal survival rate, and their therapeutic choices are restricted [32, 33]. This dilemma highlights the urgent need to explore the new mechanisms of cervical cancer development and to develop more effective therapeutic strategies. A recent study analyzed the efficacy of different therapeutic regimens for locally advanced or recurrent/metastatic cervical cancer, and the results suggest that immunotherapy and targeted therapies have shown encouraging therapeutic potential and survival benefits in some cervical cancer patients [34]. This suggests that in‐depth analysis of TIME and its regulatory mechanisms in cervical cancer is a key direction for the discovery of new therapeutic targets and optimization of existing therapies. In this study, we deeply explored the status and role of cellular pyroptosis‐related molecules in the immune microenvironment of cervical cancer and their association with the disease process. A key immune checkpoint gene, LAG3, was successfully screened and identified, which provides an important theoretical basis and a new idea for the development of novel immunotherapeutic drugs based on the LAG3 target (e.g., LAG3 inhibitor monotherapy or combination therapy) for cervical cancer.

Pyroptosis is an inflammatory form of PCD triggered by disrupted cellular homeostasis [13], activation of the caspase family is particularly important for the onset of pyroptosis [14]. There is a strong correlation between the incidence of pyroptosis and the formation of cancers. Furthermore, the production of IL‐18, which is mediated by the Nlrp3 inflammasome, has the potential to suppress the growth of colon tumors effectively [35]. Activation of the pyroptotic pathway increases the secretion of IL‐1β, which can effectively inhibit the growth of melanoma [36]. Induction of IL‐1β release in nasopharyngeal carcinoma potently enhances antitumor effects [37]. However, pyroptosis can inhibit tumorigenesis by releasing inflammatory factors, and at the same time, it also reduces the sensitivity of the body to tumor cells, thereby promoting tumor development [38, 39]. Activation of the essential protein GSDME of the pyroptosis pathway has the potential to effectively increase the sensitivity of cisplatin in lung cancer [40]. Furthermore, the expression of genes associated with pyroptosis is also highly connected with the responsiveness of chemotherapy medicines used to treat colon cancer [41]. It can be seen that pyroptosis is a potential research direction for cancer treatment.

Our study provides insight into the immunological status of cervical cancer cells with pyroptosis based on PRG molecular typing. Although cellular pyroptosis has been characterized in colorectal [41] and ovarian [42] cancers, we revealed a pattern specific to cervical cancer. The immune activation state of PRG cluster A with IL‐1β/IL‐18 release associated with pyroptosis is consistent with studies of antitumor immunity [43]. Notably, although LAG3 is recognized as an immune checkpoint in melanoma and lung cancer [44], we demonstrated by cell function experiments that LAG3 inhibited the proliferation of cervical cancer cells. This dual role of immunomodulation and intrinsic tumor suppression makes LAG3 distinct from PD‐1/CTLA‐4 and may explain the association of anti‐PD‐1 resistance with LAG3/FGL1 co‐expression [45].

### 3.2 Mechanistic Insights Into LAG3 Function

Our study demonstrated that *LAG3* knockdown enhanced tumor proliferation and migration, while inhibiting E‐cadherin and activating cellular pyroptosis effectors. Single‐cell analysis confirmed that LAG3 was expressed predominantly in Treg and CD8^+^ T cells, suggesting a role in suppressing antitumor immunity, consistent with the known inhibition of TCR signaling [46]. In addition, LAG3 may interfere with the Wnt/EMT pathway by inhibiting β‐catenin and N‐cadherin, which is further supported by the enrichment of “T‐cell activation” in LAG3‐overexpressing samples in GO and KEGG analyses. However, although *LAG3* knockdown upregulated GSDMD‐N, the associated functional impact remains to be validated. We speculate that LAG3 may regulate inflammatory vesicle activation via IL‐1β feedback [37], a pathway that warrants further investigation in the future given the dual role of IL‐1β in tumor promotion and suppression.

### 3.3 Clinical Translation and Therapeutic Implications

We constructed a risk score model based on 5 key genes. The sensitivity and specificity of this prognostic model in short‐term (1 year) prognosis were high (AUC > 0.85), while the AUC at 3 and 5 years was stable between 0.65 and 0.70, which indicated its predictive value for medium‐term and long‐term survival. The clinical application of the model is mainly in risk stratification and treatment selection. Low‐risk patients showed sensitivity to PD‐1 inhibitors, and combining LAG3 modulation with PD‐1 inhibition may help overcome resistance [45]. In contrast, high‐risk patients exhibited chemoresistance, suggesting that LAG3 agonists may work synergistically with existing treatment regimens. Further, our findings position LAG3 as a therapeutic target, which provides a rationale for developing LAG3 agonists in cervical cancer.

### 3.4 Limitations of the Study

Although this study provides new insights into the prognosis and treatment of PRGs in cervical cancer, there are some limitations. First, the small sample sizes of the TCGA and GEO datasets may limit the statistical power of the molecular typing and risk score models and their generalizability. Second, although our subcutaneous tumor model showed that *LAG3* knockdown promoted tumor growth, the mouse immune system and tumor microenvironment differed significantly from that of humans, and these interspecies differences may require further validation of the clinical relevance of LAG3 through patient‐derived xenograft (PDX) models or clinical cohorts. In addition, although we found an inhibitory role for LAG3 in proliferation and migration, the specific molecular mechanism between it and the cellular pyroptosis pathway remains unclear. Further, the predicted chemotherapy sensitivity based on IC50 values analyzed from the GDSC database reflects the in vitro drug response but may not be fully representative of the clinical outcome. Finally, due to the limited number of human samples, this study requires further prospective studies integrating data from real‐world studies to refine the application of risk score models in personalized therapy.

In conclusion, we comprehensively analyzed and demonstrated the crucial role played by pyroptosis and immunomodulation in cervical cancer using a multicenter dataset. In addition, we constructed a prognostic prediction model by combining key genes and clinicopathological parameters and found an important role for LAG3 in immunomodulation and tumor suppression in cervical cancer.

## 4 Methods

### 4.1 Cervical Cancer Data Collecting and Processing

The Cancer Genome Atlas (TCGA) database (https://portal.gdc.cancer.gov) microarray dataset and clinical information of 306 cervical cancer patients was obtained. We gathered cervical cancer somatic mutations and CNV data from the University of California Santa Cruz (UCSC) database (https://xena.ucsc.edu/). We computed the asymmetric tumor mutational burden (TMB) per megabase for each sample based on the total number of mutations found by the exome sequencing technique. The GSE30759 microarray data, which includes 48 cervical cancer cases, and the single‐cell datasets GSE171894 and GSE168652 were collected from the Gene Expression Omnibus (GEO) database (https://www.ncbi.nlm.nih.gov/geo/). The TCGA microarray dataset (FPKM) was normalized and converted to transcripts per million to maintain consistency between TCGA and GEO data (TPM). The data was processed using Perl (version 5.30.1) and R (version 4.1.2).

### 4.2 Consensus Clustering Identifies Patterns of PRGs

Based on earlier evaluations [42, 47, 48, 49, 50, 51, 52, 53, 54, 55, 56], 52 PRGs were discovered (Table S1). In normal and tumor tissues, differentially expressed genes (DEGs) were identified using the “limma” program (logFC > 1, *p* < 0.05). We utilized the R package “Consensus Cluster Plus” to conduct unsupervised clustering and identify molecular subtypes of the data based on the expression levels of the 52 PRGs; the consensus clustering mode was set to *K* = 2–9, and the optimal mode was selected, cycled 1000 times to ensure the results were stable. The “ggplot2” package was used to conduct PCA analysis, which indicated the independence of various typed groupings.

### 4.3 Functional Recognition and Immune Characterization of Gene Clusters

MSigDB's “c2.cp.kegg.v7.4.symbols.gmt” file was used to investigate PRG functions in biological pathways. Subsequently, Gene Set Variation Analysis (GSVA) was conducted using the “GSEABase” and “GSVA” packages, where |log2FC| > 0.1 and adjusted *p* < 0.05 were considered significantly enriched. Immune cell subpopulations were characterized using the genomic signature established by Charoentong et al., which provides standardized genetic markers for 23 human immune cell phenotypes in the tumor microenvironment [57]. Quantitative calculation of the level of immune cell infiltration was achieved by the ssGSEA (Single Sample Gene Set Enrichment Analysis) algorithm. The stromal score, immune score, estimate score, and immune cell purity were calculated using the “estimate” and “CIBERSORT” packages. Gene Ontology (GO) was used to annotate gene functions, while the Kyoto Encyclopedia of Genes and Genomes (KEGG) was used to examine gene‐related signaling pathways.

### 4.4 Construction and Evaluation of Risk Scoring Prediction Model

As a measure of randomness, the TCGA and GEO datasets were pooled and randomly split into the training group (177 cases) and the testing group (177 cases) using SRS. Univariate Cox regression analysis was used to evaluate the expression value of each DEG. Multivariate Cox regression analysis was then used to determine the top 5 DEGs with the best prediction performance. These DEGs include GNAZ, LAG3, IL‐1β, CA2, and SPRR3. To create risk rating models, LASSO regression analysis was performed. The testing group was used to verify the training group's results. The risk score for each patient was calculated as follows: (Exp gene1 × coefficient gene1) + (Exp gene2 × coefficient gene2) + … + (Exp gene5 × coefficient gene5).

Patients were divided into two groups with high and low risk scores based on the median of risk assessments in the training group. The Kaplan–Meier curve was used to illustrate the patient's OS, and the model's accuracy was assessed using the AUC of the ROC curve to determine the model's predictive capacity.

### 4.5 Construction and Performance Evaluation of Nomogram

Based on the clinicopathological data of cervical cancer patients, the R package “rms” was used to build a nomogram; the performance of the nomogram was evaluated by comparing the 1‐, 3‐, and 5‐year OS calibration curves.

### 4.6 Drug Susceptibility Prediction

Using data from the Genomics of Drug Sensitivity in Cancer (GDSC) database (https://www.cancerrxgene.org/), the “pRRophetic” package was used to calculate the maximal inhibitory concentration.

### 4.7 Cell Culture and Transfection

American Type Culture Collection (ATCC, USA) provided and identified the Hela and Siha cells utilized in this study. Hela and Siha cells were cultured in DMEM media supplemented with 10% fetal bovine serum. All cell lines were negative for mycoplasma infection. For transfection, Lipofectamine 3000 (L3000015, Invitrogen) was utilized.

### 4.8 Cell Phenotype Assay

To detect cell viability in the CCK8 assay, 3000 transfected cells were inoculated into each well of a 96‐well plate. Subsequently, each well was injected with 10 μL of CCK‐8 solution (HY‐K0301, MCE) at different time intervals, and the absorbance at 450 nm was recorded.

The transfected cells were cultured in 6‐well plates at a density of 1000 cells per well for the colony formation assay and incubated at 37°C for about 2 weeks. After discarding the medium, the cells were fixed with 4% formaldehyde for 30 min, stained with 0.1% crystal violet for 30 min, rinsed with water, and dried. The cells were counted using ImageJ software.

In the transwell assay, 200 μL of serum‐free medium was combined with 40,000 cells, which were then injected into the top chambers with or without matrix gel (Corning, USA). Subsequently, 600 μL of serum‐containing medium was filled into the bottom chambers. Cells were incubated for 24 h, fixed for 30 min, and then stained with crystal violet for 1 h. After washing and drying, the cells were counted using ImageJ software.

### 4.9 Western Blot Analysis

Cells were added to RIPA buffer including phosphatase inhibitors and protease inhibitors, lysed on ice for 30 min, and then the proteins were denatured at 100°C. After separation on SDS‐PAGE gels, denatured proteins were transferred to PVDF membranes. The PVDF membrane was soaked with 5% skimmed milk and incubated with a specific primary antibody and a matched secondary antibody. Finally, protein levels were detected by chemiluminescence.

### 4.10 Immunofluorescence

The cells are positioned on a 6‐well plate with sterile 22 × 22 mm coverslips. The cellular concentration was modified to around 30% during 24 h. The cells were immobilized using a 4% paraformaldehyde solution for 20 min. Subsequently, they were rinsed thrice with PBS and subjected to a 0.4% Triton X‐100 treatment for 10 min. The cells were rinsed thrice with PBS. The sections were obstructed using a 2% BSA solution for 30 min, followed by two washes with PBS. Introduce the pre‐set primary antibody and allow it to incubate at a temperature of 4°C for the night. The secondary antibody was introduced and allowed to undergo incubation for 1 h at ambient temperature. Subsequently, a concentration of 0.5 μg/mL DAPI, produced in PBS, was added and left for 10 min. The acquisition of images was performed using a laser confocal microscope (C2, Nikon, Japan).

### 4.11 Human Tissues

Eight cervical cancer tissues and 8 corresponding paraneoplastic tissues were obtained from Zhongnan Hospital, Wuhan University, Department of Gynecologic Oncology. The research received permission from the Institutional Ethics Review Board (approval number: 2020029).

### 4.12 Animal Study

Animal experiments were approved by the Zhongnan Hospital of Wuhan University's Experimental Animal Welfare Ethics Committee (Approval No. ZN2024081). We bought female BALB/c mice that were 4 weeks old from GemPharmatech Co. Ltd. (Jiangsu, China). Lentiviral transfection of U14 cells was used in animal experiments. Genpharma (Shanghai, China) supplied the lentivirus, and puromycin (1 μg/mL, Sigma) was used to screen the transfection.

In the subcutaneous tumor‐bearing model, mice were randomly divided into control (*shNC*) and experimental (*shLAG3*) groups by an independent investigator who was not involved in the subsequent experimental procedure. Mice were injected with 1 × 10^7^ U14 cells per subcutaneous injection site. The tumor size was evaluated using the following formula, which was recorded every 3 days starting from the second week following the injection: tumor size (mm^3^) = (length×width^2^)/2. Tumor volume measurements and endpoint analyses were performed by researchers who were unaware of the grouping of mice to ensure that the study process was blinded and statistical analyses were performed before unblinding.

### 4.13 Single Cell Data Analysis

We used the “harmony” R package for data integration of cervical cancer samples from GSE168652 and GSE171894. Quality control of the sample data was performed with screening criteria of UMI counts between 400 and 20,000 per cell, number of characterized genes between 300 and 6,000, and proportion of mitochondrial genes below 20%. Next, the gene expression data were normalized using the “LogNormalize” method, and then the “vst” method was used to filter out highly variable genes. In order to minimize the interference of cell cycle on the results, we applied the “CellCycleScoring” function to cycle score the cells, thus removing the potential influence of cell cycle in the analysis. For cell clustering, we chose 30 principal components and set the clustering resolution to 0.3 to obtain a reasonable cell population. In order to facilitate the visualization of the cell population, the “Uniform Mobility Approximation and Projection (UMAP)” method was adopted for nonlinear dimensionality reduction. For cell type annotation, we used the “SingleR” R package to annotate each cell cluster with known marker genes. Primary cell clusters were found using the “primitive Louvain algorithm”, and differentially expressed genes were identified by the “FindAllMarkers” function, which was then utilized for trajectory inference. The trajectory skeleton was constructed by dimensionality reduction using the “reduceDimension” function. The “orderCells” function was used to sort the cells, calculate the position and pseudo‐time value of the cells on the trajectory, and display the trajectory map through the “plot_cell_trajectory” function.

### 4.14 Statistical Analysis

The “limma” package was used to conduct univariate and multivariate Cox regression analyses to provide independent predicted values. LASSO‐Cox regression analysis with 10‐fold cross‐validation was conducted using the “glmnet” package. Using the “survival” package, we generated Kaplan–Meier survival curves and calculated log‐rank tests. The “rms” and “timeROC” packages were used to create calibration and ROC curves, respectively. The “GSVA” software was used to compare genomes. OS was calculated using Kaplan–Meier curves. R software (version 4.1.2) and the Perl programming language were used to conduct all statistical analyses (version 5.30.1).

## Author Contributions

**Shijie Yao:** conceptualization. **Siming Chen:** data curation. **Shimeng Wan:** methodology. **Anjin Wang:** supervision. **Ziyan Liang:** writing – review and editing. **Xuelian Liu:** investigation. **Yang Gao:** project administration. **Hongbing Cai:** funding acquisition.

## Ethics Statement

All research involving human participants strictly adhered to the principles outlined in the Declaration of Helsinki and has been approved by the Ethics Committee of Zhongnan Hospital of Wuhan University (Approval number: 2020029), and written informed consent was obtained from all patients. Animal experiments were approved by the Zhongnan Hospital of Wuhan University's Experimental Animal Welfare Ethics Committee (Approval No. ZN2024081).

## Conflicts of Interest

The authors declare no conflicts of interest.

## Supporting information


**Data S1.** cam471278‐sup‐0001‐DataS1.docx.

## Data Availability

The data are available from the TCGA database (https://portal.gdc.cancer.gov/), the GEO database (http://www.ncbi.nlm.nih.gov/geo/).
