# Supplementary material for: LAG3 as a Tumor Suppressor and Immune Regulator in Cervical Cancer: From Functional Validation to Therapeutic Strategy
Source: Cancer Med. 2025 Sep 30;14(19):e71278. doi: 10.1002/cam4.71278 (PMC12481447; doi:10.1002/cam4.71278)
Supplement: Supplementary file 1 — Data S1. cam471278‐sup‐0001‐DataS1.docx. [file CAM4-14-e71278-s001.docx]

**Supplementary Information**

**LAG3 as a Tumor Suppressor and Immune Regulator in Cervical Cancer: From Functional Validation to Therapeutic Strategy**

Figure S1-S11: Pages 2-18

Table S1-S5: Pages 19-31

**Figure S1-S11**

**Supplementary Figure 1**. **Cross-linked network and prognosis of pyroptosis-related genes.** (A) Correlation network of PRGs in TCGA dataset and GEO dataset in cervical cancer cohort. (B-L) The Kaplan-Meier curves of 11 PRGs demonstrated a favorable correlation with the prognosis of cervical cancer in both the TCGA dataset and GEO dataset.

**Supplementary Figure 2**. **OS Kaplan-Meier curves of 11 PRGs negatively correlated with cervical cancer prognosis in cervical cancer samples from TCGA dataset and GEO dataset.**

**Supplementary Figure 3**. **Consensus clustering based on pyroptosis-related genes.** (A-G) Matrix heatmap of cervical cancer with K=2-9. (H) Consensus index based on cumulative distribution of pyroptosis-related gene expression. (I) The range under the consensus clustering curve for K=2-9. (J) Analyze the overall survival (OS) using Kaplan-Meier method for two gene clusters. (K) PCA analysis between the two genotypes.

**Supplementary Figure 4.** **Functional analysis of DEGs among pyroptosis-related gene clusters.** (A) A GO enrichment analysis was performed on a set of 324 genes that showed differential expression. (B) KEGG enrichment analysis was conducted on 324 differentially expressed genes (DEGs).

**Supplementary Figure 5**. **Clustering based on DGE consensus.** (A-H) Matrix heatmap of cervical cancer with K=2-9. (I) Cumulative distribution consensus index based on DEG expression. (J) The range under the consensus clustering curve for K=2-9. (K) PCA analysis between the 4 genotypes.

**Supplementary Figure 6**. **Building a risk score based on DEG clusters.** (A) Distribution of patients in all cervical cancer patient cohorts according to the training group median risk score. (B) Survival outcomes of individual patients in all cohorts of cervical cancer patients. The low-risk score group is positioned to the left of the dotted line, while the high-risk score group is positioned to the right of the dotted line. (C) Heatmap of the distribution of 5 key DEGs involved in risk score construction across all cervical cancer patient cohorts. (D) In the train group, the distribution of patients is examined. (E) Survival status of each patient in the train group column. The low-risk score group is located on the left side of the dotted line, while the high-risk score group is located on the right side. (F) Across the train group, a heatmap shows the distribution of the 5 key DEGs involved in risk score construction. (G) Distribution of patients in the test group based on median risk scores. (H) Test group survival outcome for low-risk and high-risk patients. Low-risk patients are positioned to the left of the dotted line, and high-risk patients are positioned to the right. (I) Heatmap showing the distribution of the 5 key DEGs involved in risk score construction across the test group. (J) Performing a Kaplan-Meier survival analysis to compare the survival rates of high-risk and low-risk groups in all cohorts of cervical cancer patients. (K) ROC curves of the prediction model in all cervical cancer patient cohorts.

**Supplementary Figure 7**. **Forecasting the survival rate of cervical cancer using a nomogram.** (A) A nomogram is provided for the purpose of forecasting the survival rate of patients with cervical cancer. (B) Calibration curves for nomograms used to forecast 1-, 3-, and 5-year overall survival.

**Supplementary Figure 8**. **Risk score and the prediction of chemotherapeutic drug responsiveness, as well as the variation in chemotherapeutic drug IC50 across groups with high and low risk scores.**

**Supplementary Figure 9**. **Overall survival of key genes in cervical cancer.** (A) The expression of LAG3 in cervical cancer is significantly correlated with prognosis (*p* = 0.0027). (B) The expression of IL1B in cervical cancer is significantly correlated with prognosis (*p* = 0.0014). (C) The expression of CA2 was not shown to be correlated with the prognosis of cervical cancer (*p* = 0.17). (D) The expression of GNAZ was not shown to be connected with the prognosis of cervical cancer (*p* = 0.14). (E) The expression of SPRR3 did not show any link with the prognosis of cervical cancer (*p* = 0.17).

**Supplementary Figure 10**. **Dose optimization and validation of overexpression efficiency of LAG3 in cervical cancer cells.** (A) Validation of *siLAG3* transfection dose response and LAG3 plasmid overexpression efficiency in Hela and Siha cells using Western Blot analysis. (B) Validation of *siLAG3* transfection dose response and LAG3 plasmid overexpression efficiency in Hela and Siha cells using RT-qPCR analysis. The one-way ANOVA with Dunnett's multiple comparisons test was utilized to ascertain statistical differences within the LAG3 knockdown group, the two-tailed unpaired Student's t-test was used to assess the statistical difference for the LAG3 overexpression group (B). (ns, not statistically significant; ***p* < 0.01; ****p* < 0.001).

**Supplementary Figure 11.** **Analysis of functional enrichment.** (A) Performing GO enrichment analysis on LAG3. (B) Performing KEGG enrichment analysis on LAG3.

**Table S1-S5**

**Table S1. 52 pyroptosis-related genes from prior reviews.**

BAK1

BAX

CASP1

CASP3

CASP4

CASP5

CHMP2A

CHMP2B

CHMP3

CHMP4A

CHMP4B

CHMP4C

CHMP6

CHMP7

CYCS

ELANE

GSDMD

GSDME

GZMB

HMGB1

IL18

IL1A

IL1B

IRF1

IRF2

TP53

TP63

AIM2

CASP6

CASP8

CASP9

GPX4

GSDMA

GSDMB

GSDMC

IL6

NLRC4

NLRP1

NLRP2

NLRP3

NLRP6

NLRP7

NOD1

NOD2

PJVK

PLCG1

PRKACA

PYCARD

SCAF11

TIRAP

TNF

GZMA

**Table S2. 324 DEGs associated with cluster A and cluster B.**

| gene | logFC | pValue | adj.P.Val |
| --- | --- | --- | --- |
| AIM2 | -3.2507 | 7.28E-73 | 8.16E-69 |
| CASP1 | -1.20987 | 4.68E-34 | 1.75E-30 |
| CXCL10 | -2.41164 | 5.78E-32 | 1.62E-28 |
| TAP1 | -1.20787 | 1.33E-31 | 2.99E-28 |
| BNC1 | -2.48703 | 1.67E-31 | 3.13E-28 |
| CXCL11 | -2.23627 | 2.66E-31 | 4.26E-28 |
| ANXA8 | -2.15817 | 3.09E-31 | 4.34E-28 |
| GBP1 | -1.58637 | 5.03E-31 | 6.27E-28 |
| PDCD1LG2 | -1.15719 | 5.86E-31 | 6.57E-28 |
| RAC2 | -1.11829 | 6.68E-31 | 6.82E-28 |
| GBP5 | -1.84681 | 6.33E-30 | 5.92E-27 |
| HES6 | 1.61125 | 3.75E-29 | 3.24E-26 |
| GPR68 | -1.5466 | 6.38E-29 | 5.11E-26 |
| PPP1R9A | 1.206519 | 1.35E-28 | 1.01E-25 |
| CLDN3 | 3.007778 | 3.45E-28 | 2.42E-25 |
| GZMB | -1.636 | 4.16E-28 | 2.60E-25 |
| MAPK8IP1 | 1.282575 | 1.59E-27 | 9.38E-25 |
| CD274 | -1.40319 | 1.84E-27 | 1.03E-24 |
| GBP4 | -1.58268 | 2.18E-27 | 1.17E-24 |
| GNA15 | -1.27281 | 9.82E-27 | 4.77E-24 |
| ASRGL1 | 1.779674 | 1.02E-26 | 4.77E-24 |
| ATP7B | 1.491447 | 1.16E-26 | 5.17E-24 |
| CLEC2B | -1.75262 | 1.20E-26 | 5.17E-24 |
| PRF1 | -1.41793 | 1.37E-26 | 5.70E-24 |
| DNALI1 | 1.935259 | 1.54E-26 | 6.17E-24 |
| OAS2 | -1.3518 | 4.05E-26 | 1.57E-23 |
| HAVCR2 | -1.07589 | 5.46E-26 | 2.04E-23 |
| TRIM2 | 1.432959 | 1.77E-25 | 6.42E-23 |
| TSPAN12 | 1.447254 | 3.08E-25 | 1.08E-22 |
| IFIT3 | -1.42179 | 3.41E-25 | 1.16E-22 |
| APOL3 | -1.17491 | 5.55E-25 | 1.83E-22 |
| SOX15 | -2.00183 | 2.50E-24 | 7.51E-22 |
| SLAMF7 | -1.25639 | 2.54E-24 | 7.51E-22 |
| DDAH1 | 1.455042 | 3.03E-24 | 8.50E-22 |
| CCL4 | -1.19862 | 3.18E-24 | 8.70E-22 |
| KRT6A | -3.20971 | 8.53E-24 | 2.23E-21 |
| PLEKHB1 | 1.378801 | 1.54E-23 | 3.85E-21 |
| GNLY | -1.53328 | 1.54E-23 | 3.85E-21 |
| GPR87 | -2.18388 | 2.14E-23 | 5.11E-21 |
| TGM1 | -2.2241 | 2.75E-23 | 6.42E-21 |
| IL2RB | -1.14264 | 3.13E-23 | 7.16E-21 |
| ACP5 | -1.05239 | 3.23E-23 | 7.24E-21 |
| SORL1 | 1.145421 | 3.34E-23 | 7.31E-21 |
| S100A9 | -2.62745 | 3.54E-23 | 7.35E-21 |
| IFI16 | -1.24455 | 3.70E-23 | 7.54E-21 |
| GNAZ | 1.10079 | 4.59E-23 | 8.87E-21 |
| KRT5 | -3.17937 | 6.00E-23 | 1.12E-20 |
| KRT14 | -4.00807 | 7.43E-23 | 1.37E-20 |
| TUBB6 | -1.30523 | 1.08E-22 | 1.86E-20 |
| CXCL9 | -1.97245 | 1.14E-22 | 1.91E-20 |
| CCL5 | -1.44514 | 1.50E-22 | 2.43E-20 |
| DEGS2 | 1.583224 | 1.65E-22 | 2.65E-20 |
| PPP1R1B | 2.251942 | 2.13E-22 | 3.18E-20 |
| CD3E | -1.18784 | 2.51E-22 | 3.69E-20 |
| FAM83A | -1.95224 | 2.53E-22 | 3.69E-20 |
| GPR160 | 1.260703 | 2.98E-22 | 4.28E-20 |
| C1QC | -1.40222 | 4.25E-22 | 5.96E-20 |
| GZMA | -1.41331 | 4.97E-22 | 6.81E-20 |
| DACH1 | 1.218916 | 5.62E-22 | 7.59E-20 |
| SH3BGRL2 | 1.327944 | 1.09E-21 | 1.45E-19 |
| S100A8 | -2.98028 | 1.18E-21 | 1.55E-19 |
| PIP5K1B | 1.174598 | 1.21E-21 | 1.58E-19 |
| C3AR1 | -1.00626 | 1.24E-21 | 1.59E-19 |
| ADA | -1.04353 | 1.26E-21 | 1.61E-19 |
| KLHDC7B | -2.1241 | 1.32E-21 | 1.67E-19 |
| CD3D | -1.2717 | 1.44E-21 | 1.79E-19 |
| DSG3 | -2.84962 | 1.62E-21 | 1.99E-19 |
| CAPS | 1.458336 | 2.08E-21 | 2.54E-19 |
| SPATA6 | 1.004836 | 2.55E-21 | 3.01E-19 |
| PRKAA2 | 1.059873 | 2.59E-21 | 3.02E-19 |
| GZMH | -1.40158 | 2.93E-21 | 3.39E-19 |
| CSF1R | -1.05976 | 3.02E-21 | 3.45E-19 |
| STXBP6 | 1.249148 | 3.62E-21 | 4.10E-19 |
| GBP2 | -1.03313 | 3.91E-21 | 4.39E-19 |
| IFNG | -1.05739 | 4.12E-21 | 4.58E-19 |
| SLAMF8 | -1.06451 | 4.30E-21 | 4.73E-19 |
| GJB5 | -1.60262 | 4.46E-21 | 4.85E-19 |
| TRIM22 | -1.10785 | 5.98E-21 | 6.39E-19 |
| CMTM8 | 1.155951 | 6.56E-21 | 6.94E-19 |
| MSN | -1.04119 | 6.83E-21 | 7.16E-19 |
| AMOT | 1.461969 | 9.43E-21 | 9.62E-19 |
| CDH3 | -1.15688 | 1.08E-20 | 1.07E-18 |
| CD2 | -1.17656 | 1.97E-20 | 1.81E-18 |
| CD53 | -1.04951 | 3.03E-20 | 2.74E-18 |
| DSC3 | -2.67937 | 4.27E-20 | 3.84E-18 |
| FOXA2 | 1.546365 | 4.48E-20 | 3.99E-18 |
| CAPN5 | 1.379089 | 5.36E-20 | 4.70E-18 |
| C1R | -1.04294 | 5.87E-20 | 5.01E-18 |
| IL1A | -1.69661 | 7.45E-20 | 6.24E-18 |
| SLC6A20 | 1.1795 | 7.66E-20 | 6.37E-18 |
| SERPINB13 | -2.6114 | 9.13E-20 | 7.46E-18 |
| FCER1G | -1.04321 | 1.39E-19 | 1.09E-17 |
| CXCL13 | -1.78041 | 1.43E-19 | 1.11E-17 |
| PLS1 | 1.198615 | 1.52E-19 | 1.17E-17 |
| IL12RB2 | -1.1651 | 1.90E-19 | 1.43E-17 |
| GLRB | 1.065344 | 2.64E-19 | 1.96E-17 |
| TMEM125 | 1.272161 | 2.99E-19 | 2.19E-17 |
| PROM1 | 1.835705 | 3.03E-19 | 2.21E-17 |
| EHD2 | -1.00301 | 3.55E-19 | 2.56E-17 |
| CD7 | -1.11225 | 3.76E-19 | 2.67E-17 |
| TMEM40 | -1.53576 | 3.94E-19 | 2.78E-17 |
| ZNF683 | -1.25711 | 4.15E-19 | 2.91E-17 |
| EPS8 | 1.077755 | 4.82E-19 | 3.29E-17 |
| CCL3 | -1.04441 | 5.00E-19 | 3.40E-17 |
| ASB2 | -1.04435 | 6.21E-19 | 4.12E-17 |
| ICAM1 | -1.05543 | 6.95E-19 | 4.56E-17 |
| APOL1 | -1.35879 | 8.43E-19 | 5.40E-17 |
| FSCN1 | -1.41161 | 9.08E-19 | 5.75E-17 |
| SFN | -1.33531 | 1.01E-18 | 6.33E-17 |
| CHST6 | 1.346866 | 1.04E-18 | 6.42E-17 |
| GJB1 | 1.625944 | 1.05E-18 | 6.46E-17 |
| ALDH1A1 | 1.94965 | 1.12E-18 | 6.85E-17 |
| C1QA | -1.3235 | 1.28E-18 | 7.76E-17 |
| CLIC6 | 1.991148 | 1.37E-18 | 8.26E-17 |
| B4GALNT3 | 1.107726 | 1.44E-18 | 8.60E-17 |
| SNAI2 | -1.34333 | 1.47E-18 | 8.71E-17 |
| EPSTI1 | -1.10127 | 1.50E-18 | 8.85E-17 |
| RGL3 | 1.618359 | 1.77E-18 | 1.03E-16 |
| PLCB4 | 1.238278 | 3.29E-18 | 1.86E-16 |
| PEG10 | 1.780683 | 3.94E-18 | 2.19E-16 |
| CPA4 | -1.70745 | 3.94E-18 | 2.19E-16 |
| TNS4 | -1.5208 | 3.96E-18 | 2.19E-16 |
| ZMYND10 | 1.190855 | 3.98E-18 | 2.19E-16 |
| SERPING1 | -1.16563 | 4.33E-18 | 2.37E-16 |
| LAG3 | -1.10291 | 4.88E-18 | 2.66E-16 |
| DCDC2 | 1.168765 | 5.17E-18 | 2.79E-16 |
| C1QB | -1.40527 | 5.63E-18 | 3.02E-16 |
| NKG7 | -1.32759 | 5.89E-18 | 3.15E-16 |
| CLCA2 | -2.5604 | 6.80E-18 | 3.57E-16 |
| AQP5 | 2.51863 | 6.87E-18 | 3.59E-16 |
| RHOV | -1.3156 | 1.13E-17 | 5.77E-16 |
| ENPP5 | 1.280022 | 1.36E-17 | 6.81E-16 |
| TRIM29 | -1.4172 | 1.36E-17 | 6.81E-16 |
| CSTA | -2.34311 | 1.36E-17 | 6.81E-16 |
| PKP1 | -2.4275 | 1.73E-17 | 8.42E-16 |
| CD40 | -1.06788 | 1.83E-17 | 8.89E-16 |
| PSMB9 | -1.00635 | 2.41E-17 | 1.15E-15 |
| RCOR2 | 1.037438 | 3.45E-17 | 1.61E-15 |
| C1S | -1.1503 | 4.22E-17 | 1.95E-15 |
| CYBB | -1.00053 | 4.54E-17 | 2.06E-15 |
| BATF2 | -1.0587 | 4.76E-17 | 2.15E-15 |
| KLHDC7A | 1.212453 | 5.11E-17 | 2.28E-15 |
| RSAD2 | -1.20329 | 6.43E-17 | 2.83E-15 |
| CRYM | 1.218985 | 7.80E-17 | 3.37E-15 |
| CTSW | -1.177 | 8.63E-17 | 3.71E-15 |
| THBD | -1.48333 | 9.77E-17 | 4.17E-15 |
| IFI44 | -1.1592 | 1.39E-16 | 5.80E-15 |
| FOXJ1 | 2.076635 | 1.48E-16 | 6.12E-15 |
| ENTPD8 | 1.177418 | 1.69E-16 | 6.87E-15 |
| TFF3 | 2.570057 | 1.91E-16 | 7.64E-15 |
| IL1B | -1.18559 | 2.30E-16 | 9.16E-15 |
| LRIG1 | 1.018297 | 2.32E-16 | 9.21E-15 |
| IL1RN | -1.44065 | 2.57E-16 | 1.02E-14 |
| OASL | -1.09186 | 2.79E-16 | 1.10E-14 |
| MLPH | 1.343226 | 3.52E-16 | 1.36E-14 |
| CAV1 | -1.12333 | 4.09E-16 | 1.56E-14 |
| FCGR3A | -1.17952 | 6.54E-16 | 2.42E-14 |
| MMP12 | -1.4736 | 6.75E-16 | 2.49E-14 |
| IL32 | -1.05494 | 7.98E-16 | 2.90E-14 |
| CD163 | -1.01429 | 1.01E-15 | 3.63E-14 |
| METTL7B | 1.236449 | 1.19E-15 | 4.18E-14 |
| FZD8 | 1.115845 | 1.51E-15 | 5.25E-14 |
| FAM107A | 1.088783 | 1.85E-15 | 6.29E-14 |
| CA2 | -1.3722 | 1.97E-15 | 6.63E-14 |
| OLR1 | -1.26462 | 2.56E-15 | 8.54E-14 |
| RHCG | -2.5825 | 2.63E-15 | 8.72E-14 |
| TEKT2 | 1.184481 | 2.79E-15 | 9.23E-14 |
| SERPINB4 | -2.15047 | 2.84E-15 | 9.34E-14 |
| CST7 | -1.00063 | 3.49E-15 | 1.12E-13 |
| ADAMDEC1 | -1.11946 | 3.98E-15 | 1.26E-13 |
| S100A2 | -1.81781 | 4.25E-15 | 1.33E-13 |
| SCGB2A1 | 2.123001 | 4.28E-15 | 1.34E-13 |
| FAT2 | -1.57087 | 6.00E-15 | 1.82E-13 |
| PNMA6A | 1.036296 | 1.17E-14 | 3.39E-13 |
| SERPINB5 | -1.60898 | 1.30E-14 | 3.70E-13 |
| PDK4 | 1.042669 | 1.56E-14 | 4.41E-13 |
| ADRA2C | 1.152361 | 1.81E-14 | 5.04E-13 |
| LYPD3 | -1.66845 | 2.02E-14 | 5.57E-13 |
| SLC44A4 | 1.804221 | 2.07E-14 | 5.68E-13 |
| VIL1 | 1.088057 | 2.31E-14 | 6.33E-13 |
| CASP14 | -2.46589 | 2.35E-14 | 6.39E-13 |
| LSP1 | -1.01147 | 2.54E-14 | 6.85E-13 |
| APOBEC3A | -1.53879 | 4.48E-14 | 1.15E-12 |
| TNNC2 | 1.1255 | 4.87E-14 | 1.24E-12 |
| GPC4 | 1.313488 | 5.00E-14 | 1.27E-12 |
| IL2RG | -1.01536 | 5.21E-14 | 1.31E-12 |
| VSNL1 | -1.55196 | 5.31E-14 | 1.33E-12 |
| EPS8L3 | 1.37921 | 6.51E-14 | 1.60E-12 |
| FAM3B | 1.506667 | 7.01E-14 | 1.70E-12 |
| KRT16 | -2.21607 | 1.05E-13 | 2.50E-12 |
| CLEC7A | -1.08817 | 1.12E-13 | 2.65E-12 |
| GBP6 | -1.89757 | 1.25E-13 | 2.92E-12 |
| CD8A | -1.06228 | 1.66E-13 | 3.81E-12 |
| FOXA3 | 1.337519 | 1.71E-13 | 3.88E-12 |
| METTL7A | 1.094483 | 1.76E-13 | 3.96E-12 |
| COL7A1 | -1.08066 | 1.86E-13 | 4.18E-12 |
| ALOX15B | -1.29594 | 1.87E-13 | 4.19E-12 |
| SLC34A2 | 1.907224 | 2.23E-13 | 4.93E-12 |
| CLDN1 | -1.48507 | 2.41E-13 | 5.30E-12 |
| AKR7A3 | 1.014079 | 2.77E-13 | 6.00E-12 |
| CTXN1 | 1.022331 | 3.26E-13 | 6.94E-12 |
| MMP9 | -1.18668 | 3.33E-13 | 7.08E-12 |
| CLGN | 1.160351 | 3.52E-13 | 7.43E-12 |
| CALML3 | -2.41294 | 3.55E-13 | 7.48E-12 |
| HOXD11 | -1.07217 | 4.12E-13 | 8.55E-12 |
| ANGPTL4 | -1.19751 | 5.07E-13 | 1.04E-11 |
| KRT6B | -2.20769 | 7.30E-13 | 1.45E-11 |
| TREM2 | -1.00041 | 8.92E-13 | 1.76E-11 |
| EMX2 | 1.073276 | 9.32E-13 | 1.83E-11 |
| KRT17 | -1.52028 | 1.08E-12 | 2.10E-11 |
| IFI44L | -1.08634 | 1.17E-12 | 2.25E-11 |
| CLDN9 | 1.043679 | 1.32E-12 | 2.52E-11 |
| INHBB | 1.043459 | 1.89E-12 | 3.50E-11 |
| EFHD1 | 1.04631 | 1.92E-12 | 3.57E-11 |
| FBXO2 | -1.25213 | 2.03E-12 | 3.74E-11 |
| SPDEF | 1.763693 | 2.15E-12 | 3.95E-11 |
| SOX7 | -1.09646 | 2.45E-12 | 4.46E-11 |
| USH1C | 1.092477 | 2.67E-12 | 4.79E-11 |
| UGT8 | 1.260145 | 3.00E-12 | 5.30E-11 |
| ADRB2 | -1.01958 | 3.15E-12 | 5.56E-11 |
| SLC2A10 | 1.034051 | 3.37E-12 | 5.91E-11 |
| AGR2 | 1.977631 | 3.61E-12 | 6.29E-11 |
| ALDH1A2 | 1.001072 | 3.81E-12 | 6.57E-11 |
| SERPINB3 | -1.94828 | 4.00E-12 | 6.88E-11 |
| LY6D | -2.035 | 4.19E-12 | 7.15E-11 |
| COL17A1 | -1.69203 | 4.76E-12 | 8.05E-11 |
| KRT6C | -2.11067 | 5.81E-12 | 9.71E-11 |
| ECHDC3 | -1.06951 | 6.69E-12 | 1.11E-10 |
| C12orf54 | -1.08677 | 8.64E-12 | 1.41E-10 |
| MUC13 | 1.846849 | 9.01E-12 | 1.46E-10 |
| CD109 | -1.00426 | 9.89E-12 | 1.60E-10 |
| SLC5A1 | 1.144861 | 9.98E-12 | 1.61E-10 |
| SNCG | -1.32986 | 1.12E-11 | 1.76E-10 |
| HOXB8 | 1.360312 | 1.12E-11 | 1.76E-10 |
| SLC40A1 | 1.05742 | 1.12E-11 | 1.76E-10 |
| TMEM45B | 1.065208 | 1.15E-11 | 1.80E-10 |
| PIGR | 2.172584 | 1.18E-11 | 1.85E-10 |
| TMC5 | 1.314163 | 1.20E-11 | 1.87E-10 |
| IFIT1 | -1.01456 | 1.51E-11 | 2.30E-10 |
| VTCN1 | 1.415965 | 2.17E-11 | 3.21E-10 |
| MSI1 | 1.024398 | 2.28E-11 | 3.36E-10 |
| BMP3 | 1.008976 | 2.43E-11 | 3.56E-10 |
| S100A12 | -1.33623 | 2.48E-11 | 3.63E-10 |
| SLC10A6 | -1.18454 | 2.51E-11 | 3.67E-10 |
| TMEM45A | -1.14189 | 2.65E-11 | 3.84E-10 |
| COL9A2 | 1.09655 | 2.68E-11 | 3.87E-10 |
| FABP5 | -1.58722 | 3.54E-11 | 5.04E-10 |
| CCL18 | -1.23347 | 5.81E-11 | 8.00E-10 |
| TSPAN8 | 1.568696 | 9.40E-11 | 1.25E-09 |
| CST6 | -1.23217 | 9.66E-11 | 1.28E-09 |
| IVL | -1.8814 | 1.08E-10 | 1.42E-09 |
| ST6GALNAC1 | 1.185818 | 1.10E-10 | 1.44E-09 |
| ALOX15 | 1.281138 | 1.67E-10 | 2.13E-09 |
| PTGES | -1.0275 | 1.99E-10 | 2.50E-09 |
| C4BPA | 1.231427 | 2.05E-10 | 2.56E-09 |
| FST | -1.31818 | 2.36E-10 | 2.93E-09 |
| GJB2 | -1.36004 | 2.48E-10 | 3.06E-09 |
| SOX17 | 1.030469 | 3.27E-10 | 3.92E-09 |
| SAA1 | -1.40301 | 3.30E-10 | 3.95E-09 |
| ALPK3 | 1.008553 | 3.30E-10 | 3.95E-09 |
| LGALS4 | 1.221663 | 3.75E-10 | 4.45E-09 |
| KRT13 | -2.41157 | 3.76E-10 | 4.46E-09 |
| FAM3D | 1.302981 | 3.86E-10 | 4.56E-09 |
| MUC5B | 1.564121 | 4.07E-10 | 4.77E-09 |
| C10orf99 | -1.89844 | 4.34E-10 | 5.06E-09 |
| TRIM54 | 1.008346 | 4.66E-10 | 5.38E-09 |
| GDF15 | 1.149415 | 5.97E-10 | 6.72E-09 |
| SERPINB2 | -1.36313 | 7.31E-10 | 8.10E-09 |
| PAX8 | 1.192659 | 7.62E-10 | 8.38E-09 |
| HOXB5 | 1.05243 | 8.12E-10 | 8.89E-09 |
| PDPN | -1.05773 | 8.48E-10 | 9.23E-09 |
| KIF12 | 1.148338 | 9.15E-10 | 9.90E-09 |
| IGFBP2 | 1.158477 | 1.09E-09 | 1.17E-08 |
| GJB6 | -1.70225 | 1.10E-09 | 1.18E-08 |
| ADH1C | 1.272374 | 1.82E-09 | 1.87E-08 |
| CLDN10 | 1.133595 | 2.12E-09 | 2.15E-08 |
| PLA2G4E | -1.08899 | 2.24E-09 | 2.26E-08 |
| FOXN1 | -1.09339 | 2.97E-09 | 2.93E-08 |
| TMPRSS2 | 1.017607 | 3.11E-09 | 3.05E-08 |
| HAS3 | -1.10482 | 3.66E-09 | 3.54E-08 |
| S100A7 | -2.14865 | 4.02E-09 | 3.85E-08 |
| SPRR2A | -2.00192 | 4.31E-09 | 4.10E-08 |
| BASP1 | -1.00645 | 5.41E-09 | 5.06E-08 |
| WFDC5 | -1.13229 | 5.58E-09 | 5.20E-08 |
| HLA-DQA2 | -1.08158 | 6.12E-09 | 5.64E-08 |
| CRABP1 | 1.022218 | 6.67E-09 | 6.11E-08 |
| CP | 1.356302 | 8.35E-09 | 7.52E-08 |
| SCGB3A1 | 1.240327 | 9.18E-09 | 8.24E-08 |
| HOXB6 | 1.062318 | 1.04E-08 | 9.21E-08 |
| FOLR1 | 1.294676 | 1.13E-08 | 9.94E-08 |
| HOXB9 | 1.085101 | 1.59E-08 | 1.37E-07 |
| KRT24 | -1.29172 | 1.67E-08 | 1.44E-07 |
| SPRR2D | -1.79422 | 2.02E-08 | 1.71E-07 |
| TMPRSS11A | -1.46126 | 2.21E-08 | 1.86E-07 |
| GLDC | -1.0384 | 2.81E-08 | 2.32E-07 |
| SCGB1D2 | 1.040861 | 3.08E-08 | 2.52E-07 |
| CDH16 | 1.016439 | 3.41E-08 | 2.77E-07 |
| DUOX2 | -1.07401 | 4.41E-08 | 3.51E-07 |
| TMPRSS11D | -1.66478 | 4.96E-08 | 3.92E-07 |
| IGFBP5 | 1.040095 | 5.10E-08 | 4.02E-07 |
| SPRR1B | -2.05389 | 5.18E-08 | 4.08E-07 |
| FGFBP1 | -1.34564 | 8.85E-08 | 6.70E-07 |
| WFDC2 | 1.539759 | 1.00E-07 | 7.53E-07 |
| SPINK1 | 1.003637 | 1.05E-07 | 7.85E-07 |
| CYP24A1 | -1.01659 | 2.29E-07 | 1.59E-06 |
| A2ML1 | -1.2746 | 3.29E-07 | 2.22E-06 |
| GPC3 | 1.094775 | 4.37E-07 | 2.88E-06 |
| KRT15 | -1.42712 | 5.07E-07 | 3.30E-06 |
| KRT23 | 1.084202 | 5.35E-07 | 3.48E-06 |
| AQP3 | -1.01944 | 6.38E-07 | 4.10E-06 |
| EFEMP1 | -1.03527 | 1.15E-06 | 7.04E-06 |
| AKR1B10 | -1.32863 | 1.17E-06 | 7.18E-06 |
| CEACAM5 | 1.409141 | 1.84E-06 | 1.07E-05 |
| MMP13 | -1.11003 | 2.09E-06 | 1.21E-05 |
| PGLYRP3 | -1.06364 | 2.41E-06 | 1.37E-05 |
| SBSN | -1.47852 | 3.83E-06 | 2.09E-05 |
| SPRR2E | -1.42801 | 1.53E-05 | 7.29E-05 |
| SPINK5 | -1.05034 | 1.75E-05 | 8.23E-05 |
| CEACAM6 | 1.130372 | 3.03E-05 | 0.000135 |
| SPRR1A | -1.5504 | 4.22E-05 | 0.000182 |
| SPRR3 | -1.38156 | 0.00022 | 0.000791 |
| LGALS7 | -1.04717 | 0.000361 | 0.001231 |
| GABRP | 1.03781 | 0.000459 | 0.001523 |
| PI3 | -1.00886 | 0.000652 | 0.002071 |

**Table S3. 31 genes identified by univariate regression.**

| Id | HR | HR.95L | HR.95H | pvalue |
| --- | --- | --- | --- | --- |
| GNAZ | 0.793571 | 0.648916 | 0.970471 | 0.024329 |
| CCL5 | 0.843165 | 0.735837 | 0.966146 | 0.01406 |
| CD3E | 0.802027 | 0.676099 | 0.951409 | 0.011356 |
| CD3D | 0.832292 | 0.713839 | 0.970401 | 0.019102 |
| GZMH | 0.829572 | 0.719298 | 0.956752 | 0.010244 |
| CDH3 | 1.314053 | 1.081091 | 1.597216 | 0.006087 |
| CD2 | 0.763294 | 0.64904 | 0.897661 | 0.001095 |
| CD53 | 0.826573 | 0.691722 | 0.987713 | 0.036081 |
| IL1A | 1.154711 | 1.033505 | 1.290131 | 0.011009 |
| CD7 | 0.770634 | 0.651928 | 0.910953 | 0.002268 |
| ZNF683 | 0.838587 | 0.716838 | 0.981014 | 0.027845 |
| FSCN1 | 1.221 | 1.041266 | 1.431757 | 0.013983 |
| B4GALNT3 | 0.837046 | 0.707367 | 0.990499 | 0.038347 |
| LAG3 | 0.790061 | 0.663582 | 0.940647 | 0.008112 |
| NKG7 | 0.823432 | 0.72002 | 0.941697 | 0.00455 |
| IL1B | 1.36613 | 1.189112 | 1.569498 | 1.05E-05 |
| FAM107A | 0.786796 | 0.641198 | 0.965456 | 0.021637 |
| CA2 | 1.133638 | 1.001317 | 1.283444 | 0.047619 |
| CST7 | 0.768316 | 0.65242 | 0.9048 | 0.001582 |
| LSP1 | 0.785654 | 0.667941 | 0.924111 | 0.00358 |
| APOBEC3A | 0.89624 | 0.805163 | 0.997618 | 0.045115 |
| IL2RG | 0.849987 | 0.7269 | 0.993918 | 0.04171 |
| FAM3B | 0.883956 | 0.790537 | 0.988414 | 0.03043 |
| CD8A | 0.777589 | 0.666534 | 0.907148 | 0.001377 |
| ANGPTL4 | 1.220503 | 1.044586 | 1.426045 | 0.012098 |
| VTCN1 | 0.885015 | 0.791527 | 0.989544 | 0.031993 |
| GJB2 | 1.162826 | 1.037444 | 1.303362 | 0.009557 |
| ADH1C | 0.890548 | 0.793762 | 0.999136 | 0.048303 |
| FOXN1 | 0.869186 | 0.770205 | 0.980887 | 0.023038 |
| SPRR3 | 0.930205 | 0.873529 | 0.990558 | 0.024086 |
| GABRP | 0.924646 | 0.85514 | 0.9998 | 0.049417 |

**Table S4. Clinicopathological characteristics of the Zhongnan Hospital cohort based on LAG3 protein level.**

| **Patient ID** | **Gender** | **Age / ys** | **Tumor Size / cm^3^** | **TNM Stage** |
| --- | --- | --- | --- | --- |
| 1 | Female | 55 | 4*3.5*3 | pT1b1N1Mx |
| 2 | Female | 54 | 5*4*1.8 | pT2bN1M |
| 3 | Female | 47 | 4.5 *4.5 *1.5 | pT2aN1Mx |
| 4 | Female | 70 | 3 *2.5 *1 | pT1b1N0Mx |
| 5 | Female | 59 | 3.0 *1.5 *0.7 | pT1N1Mx |
| 6 | Female | 54 | 2.5*2*2 | pT1b1N0Mx |
| 7 | Female | 70 | 2.9*2.5*0.5 | pT1b1N0Mx |
| 8 | Female | 66 | 2.2*1.1*0.6 | pT1N0Mx |

**Table S5. Details of the antibodies used in this study.**

| **Antibody** | **Catalog No.** | **Source** | **Dilution or amount** |
| --- | --- | --- | --- |
| LAG3 | CSB-PA012719LA01HU | CUSABIO | WB/1:1000 |
| Ki67 | A20018 | Abclonal | IF/1:100 |
| GAPDH | 60004-1-Ig | Proteintech | WB/1:50000 |
| N-cadherin | 13116 | Cell Signaling Technology | WB/1:500 |
| E-cadherin | 3195 | Cell Signaling Technology | WB/1:500 |
| β-catenin | 8480 | Cell Signaling Technology | WB/1:1000 |
| Caspase1 | 81482-1-RR | Proteintech | WB/1:5000 |
| GSDMD | 20770-1-AP | Proteintech | WB/1:5000 |
| GSDMD-N | ab215203 | Abcam | WB/1:1000 |

WB: Western blot; IF: Immunofluorescence.
